# 3-(3-Amino­phenyl­sulfon­yl)aniline

**DOI:** 10.1107/S1600536808043389

**Published:** 2009-01-08

**Authors:** Reza Ghazisaeidi, Mohammad Yousefi

**Affiliations:** a Islamic Azad University, Tehran South Branch, Tehran, Iran; b Islamic Azad University, Shahr-e-Rey Branch, Tehran, Iran

## Abstract

In the title compound, C_12_H_12_N_2_O_2_S, the aromatic rings are oriented at a dihedral angle of 79.48 (4)°. Intra­molecular C—H⋯O hydrogen bonds result in the formation of two five-membered rings with envelope conformations. In the crystal structure, inter­molecular N—H⋯O hydrogen bonds link the mol­ecules. π–π Contacts between the benzene rings, [centroid–centroid distance = 4.211 (3) Å] may further stabilize the structure.

## Related literature

For general background, see: Block (1992 ▶); Holland (1988 ▶); McMohan *et al.* (1993 ▶). For bond-length data, see: Allen *et al.* (1987 ▶).
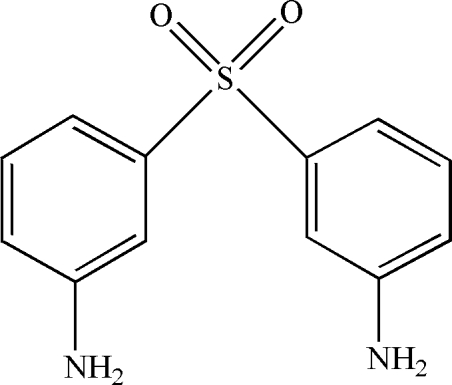


## Experimental

### 

#### Crystal data

C_12_H_12_N_2_O_2_S
*M* *_r_* = 248.30
Monoclinic, 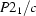

*a* = 8.6282 (17) Å
*b* = 8.8017 (18) Å
*c* = 16.052 (3) Å
β = 98.12 (3)°
*V* = 1206.8 (4) Å^3^
*Z* = 4
Mo *K*α radiation
μ = 0.26 mm^−1^
*T* = 298 (2) K
0.40 × 0.30 × 0.28 mm

#### Data collection

Bruker SMART CCD area-detector diffractometer
Absorption correction: multi-scan (*SADABS*; Bruker, 1998 ▶) *T*_min_ = 0.910, *T*_max_ = 0.933
18754 measured reflections
4145 independent reflections
2971 reflections with *I* > 2σ(*I*)
*R*_int_ = 0.091

#### Refinement

*R*[*F* ^2^ > 2σ(*F* ^2^)] = 0.078
*wR*(*F* ^2^) = 0.218
*S* = 1.12
4145 reflections
154 parameters
H-atom parameters constrained
Δρ_max_ = 0.64 e Å^−3^
Δρ_min_ = −0.26 e Å^−3^

### 

Data collection: *SMART* (Bruker, 1998 ▶); cell refinement: *SAINT* (Bruker, 1998 ▶); data reduction: *SAINT*; program(s) used to solve structure: *SHELXTL* (Sheldrick, 2008 ▶); program(s) used to refine structure: *SHELXTL*; molecular graphics: *ORTEP-3 for Windows* (Farrugia, 1997 ▶); software used to prepare material for publication: *WinGX* (Farrugia, 1999 ▶).

## Supplementary Material

Crystal structure: contains datablocks I, global. DOI: 10.1107/S1600536808043389/hk2599sup1.cif

Structure factors: contains datablocks I. DOI: 10.1107/S1600536808043389/hk2599Isup2.hkl

Additional supplementary materials:  crystallographic information; 3D view; checkCIF report

## Figures and Tables

**Table 1:** Hydrogen-bond geometry (Å, °)

*D*—H⋯*A*	*D*—H	H⋯*A*	*D*⋯*A*	*D*—H⋯*A*
N1—H1*B*⋯O2^i^	0.86	2.25	3.091 (5)	166
N2—H2*A*⋯O1^ii^	0.86	2.29	3.069 (4)	151
N2—H2*B*⋯O2^iii^	0.86	2.38	3.187 (4)	156
C1—H1⋯O2	0.93	2.55	2.924 (4)	104
C8—H8⋯O1	0.93	2.51	2.895 (3)	105

Symmetry codes: (i) 
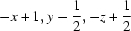
; (ii) 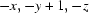; (iii) 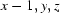.

**Fig. 1.**
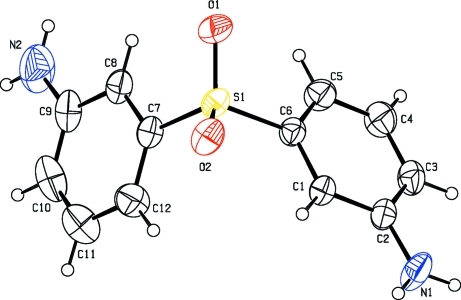
The molecular structure of the title molecule, with the atom-numbering scheme. Displacement ellipsoids are drawn at the 30% probability level.

**Fig. 2.**
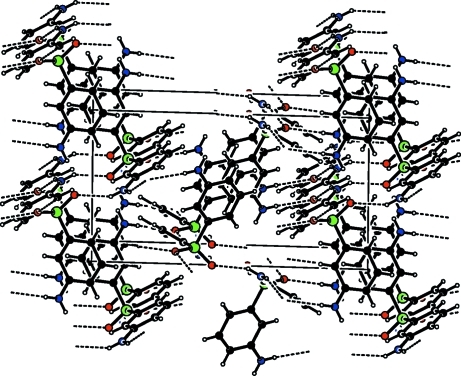
A partial packing diagram of the title compound. Hydrogen bonds are shown as dashed lines.

## supplementary crystallographic information

### Comment

Aryl sulfones and sulfoxides are interesting functional groups possessing manifold reactivity for conversion to a variety of organosulfur compounds in the fields of drugs and pharmaceuticals (Holland, 1988; Block, 1992). In particular, aryl sulfones have received much attention as powerful anti-HIV-1 agents (McMohan *et al.*,  1993). We report herein the synthesis and crystal structure of the title compound.

In the molecule of the title compound (Fig 1), the bond lengths (Allen *et al.*,  1987) and angles are within normal ranges. Rings A (C1-C6) and B (C7-C12) are, of course, planar, and they are oriented at a dihedral angle of 79.48 (4)°. The intramolecular C-H···O hydrogen bonds (Table 1) result in the formations of two five-membered rings C (S1/O1/C7/C8/H8) and D (S1/O2/C1/C6/H1), having envelope conformations with atoms O1 and O2 displaced by -0.386 (4) Å and 0.300 (4) Å, respectively, from the planes of the other ring atoms.

In the crystal structure, intermolecular N-H···O hydrogen bonds (Table 1) link the molecules (Fig. 2), in which they may be effective in the stabilization of the structure. The π-π contact between the phenyl rings, Cg1—Cg1^i^ [symmetry code: (i) 1 - x, -y, -z, where Cg1 is centroid of the ring A (C1-C6)] may further stabilize the structure, with centroid-centroid distance of 4.211 (3) Å.

### Experimental

For the preparation of the title compound, a solution of 3,3'-diaminodiphenyl sulfone (0.52 g, 2.0 mmol) in methanol (10 ml) was added to a solution of pyrazinecarboxylic acid (0.51 g, 4.0 mmol) in methanol (20 ml), and the resulting yellow solution was stirred for 40 min at 313 K. It was left to evaporate slowly at room temperature. After one week, yellow prismatic crystals of the title compound were isolated (yield; 0.45 g, 86.5%).

### Refinement

H atoms were positioned geometrically, with N-H = 0.86 Å (for NH_2_) and C-H = 0.93 Å for aromatic H and constrained to ride on their parent atoms, with U_iso_(H) = 1.2U_eq_(C,N).

### Crystal data

**Table:**

C_12_H_12_N_2_O_2_S	*F*(000) = 520
*M**_r_* = 248.30	*D*_x_ = 1.367 Mg m^−^^3^
Monoclinic, *P*2_1_/*c*	Mo *K*α radiation, λ = 0.71073 Å
Hall symbol: -P 2ybc	Cell parameters from 1532 reflections
*a* = 8.6282 (17) Å	θ = 2.4–32.0°
*b* = 8.8017 (18) Å	µ = 0.26 mm^−^^1^
*c* = 16.052 (3) Å	*T* = 298 K
β = 98.12 (3)°	Colorless, yellow
*V* = 1206.8 (4) Å^3^	0.40 × 0.30 × 0.28 mm
*Z* = 4	

### Data collection

**Table:**

Bruker SMART CCD area-detector diffractometer	4145 independent reflections
Radiation source: fine-focus sealed tube	2971 reflections with *I* > 2σ(*I*)
graphite	*R*_int_ = 0.091
φ and ω scans	θ_max_ = 32.0°, θ_min_ = 2.4°
Absorption correction: multi-scan (*SADABS*; Bruker, 1998)	*h* = −12→12
*T*_min_ = 0.910, *T*_max_ = 0.933	*k* = −12→13
18754 measured reflections	*l* = −23→23

### Refinement

**Table:**

Refinement on *F*^2^	Primary atom site location: structure-invariant direct methods
Least-squares matrix: full	Secondary atom site location: difference Fourier map
*R*[*F*^2^ > 2σ(*F*^2^)] = 0.078	Hydrogen site location: inferred from neighbouring sites
*wR*(*F*^2^) = 0.218	H-atom parameters constrained
*S* = 1.12	*w* = 1/[σ^2^(*F*_o_^2^) + (0.0789*P*)^2^ + 0.6213*P*] where *P* = (*F*_o_^2^ + 2*F*_c_^2^)/3
4145 reflections	(Δ/σ)_max_ = 0.003
154 parameters	Δρ_max_ = 0.64 e Å^−^^3^
0 restraints	Δρ_min_ = −0.26 e Å^−^^3^

### Special details

**Table:**

Geometry. All e.s.d.'s (except the e.s.d. in the dihedral angle between two l.s. planes) are estimated using the full covariance matrix. The cell e.s.d.'s are taken into account individually in the estimation of e.s.d.'s in distances, angles and torsion angles; correlations between e.s.d.'s in cell parameters are only used when they are defined by crystal symmetry. An approximate (isotropic) treatment of cell e.s.d.'s is used for estimating e.s.d.'s involving l.s. planes.
Refinement. Refinement of *F*^2^ against ALL reflections. The weighted *R*-factor *wR* and goodness of fit *S* are based on *F*^2^, conventional *R*-factors *R* are based on *F*, with *F* set to zero for negative *F*^2^. The threshold expression of *F*^2^ > σ(*F*^2^) is used only for calculating *R*-factors(gt) *etc*. and is not relevant to the choice of reflections for refinement. *R*-factors based on *F*^2^ are statistically about twice as large as those based on *F*, and *R*- factors based on ALL data will be even larger.

### Fractional atomic coordinates and isotropic or equivalent isotropic displacement parameters (Å^2^)

**Table:**

	*x*	*y*	*z*	*U*_iso_*/*U*_eq_	
S1	0.25722 (7)	0.35241 (8)	0.12847 (4)	0.0538 (2)	
O1	0.2448 (2)	0.4691 (2)	0.06463 (15)	0.0705 (6)	
O2	0.3825 (2)	0.3662 (3)	0.19740 (15)	0.0734 (6)	
N1	0.4728 (5)	−0.1878 (5)	0.1176 (3)	0.1187 (14)	
H1A	0.4757	−0.2745	0.0933	0.142*	
H1B	0.5288	−0.1722	0.1656	0.142*	
N2	−0.3243 (3)	0.4789 (4)	0.1136 (2)	0.0844 (9)	
H2B	−0.4113	0.4778	0.1340	0.101*	
H2A	−0.3183	0.5252	0.0670	0.101*	
C1	0.3714 (3)	0.0656 (3)	0.11930 (19)	0.0633 (7)	
H1	0.4308	0.0854	0.1711	0.076*	
C2	0.3800 (4)	−0.0758 (4)	0.0805 (2)	0.0765 (9)	
C3	0.2912 (6)	−0.1002 (5)	0.0046 (3)	0.0977 (13)	
H3	0.2966	−0.1946	−0.0208	0.117*	
C4	0.1948 (6)	0.0077 (5)	−0.0357 (3)	0.0979 (13)	
H4	0.1368	−0.0127	−0.0879	0.117*	
C5	0.1844 (5)	0.1493 (4)	0.0026 (2)	0.0785 (9)	
H5	0.1187	0.2244	−0.0234	0.094*	
C6	0.2733 (3)	0.1754 (3)	0.07936 (18)	0.0558 (6)	
C7	0.0781 (3)	0.3441 (3)	0.16923 (16)	0.0498 (5)	
C8	−0.0511 (3)	0.4117 (3)	0.12433 (16)	0.0513 (5)	
H8	−0.0433	0.4605	0.0737	0.062*	
C9	−0.1947 (3)	0.4063 (3)	0.15552 (18)	0.0545 (6)	
C10	−0.2009 (4)	0.3306 (4)	0.2309 (2)	0.0645 (7)	
H10	−0.2956	0.3251	0.2522	0.077*	
C11	−0.0704 (4)	0.2637 (4)	0.2746 (2)	0.0735 (8)	
H11	−0.0780	0.2140	0.3250	0.088*	
C12	0.0725 (4)	0.2692 (4)	0.24466 (19)	0.0660 (7)	
H12	0.1613	0.2242	0.2741	0.079*	

### Atomic displacement parameters (Å^2^)

**Table:**

	*U*^11^	*U*^22^	*U*^33^	*U*^12^	*U*^13^	*U*^23^
S1	0.0398 (3)	0.0559 (4)	0.0663 (4)	−0.0023 (2)	0.0092 (2)	0.0031 (3)
O1	0.0610 (12)	0.0647 (12)	0.0897 (15)	−0.0026 (9)	0.0239 (11)	0.0198 (11)
O2	0.0473 (10)	0.0876 (15)	0.0821 (14)	−0.0077 (10)	−0.0019 (10)	−0.0096 (12)
N1	0.136 (3)	0.095 (2)	0.130 (3)	0.045 (2)	0.036 (3)	−0.002 (2)
N2	0.0493 (13)	0.108 (2)	0.101 (2)	0.0197 (14)	0.0271 (14)	0.0260 (18)
C1	0.0570 (15)	0.0685 (17)	0.0684 (17)	0.0104 (12)	0.0225 (13)	0.0047 (14)
C2	0.084 (2)	0.0656 (18)	0.088 (2)	0.0161 (16)	0.0439 (19)	0.0042 (16)
C3	0.135 (4)	0.079 (2)	0.089 (3)	−0.002 (2)	0.050 (3)	−0.020 (2)
C4	0.124 (4)	0.091 (3)	0.079 (2)	−0.004 (3)	0.018 (2)	−0.013 (2)
C5	0.083 (2)	0.082 (2)	0.0693 (19)	0.0001 (18)	0.0068 (17)	−0.0018 (17)
C6	0.0501 (12)	0.0577 (14)	0.0627 (15)	0.0024 (10)	0.0182 (11)	0.0013 (11)
C7	0.0449 (11)	0.0504 (12)	0.0552 (13)	−0.0016 (9)	0.0105 (10)	−0.0026 (10)
C8	0.0450 (11)	0.0535 (13)	0.0572 (13)	0.0022 (10)	0.0131 (10)	0.0021 (11)
C9	0.0466 (12)	0.0540 (13)	0.0652 (15)	0.0025 (10)	0.0155 (11)	−0.0061 (11)
C10	0.0601 (15)	0.0708 (18)	0.0678 (17)	−0.0050 (13)	0.0272 (13)	−0.0045 (13)
C11	0.0748 (19)	0.087 (2)	0.0623 (17)	−0.0027 (16)	0.0236 (15)	0.0147 (16)
C12	0.0609 (16)	0.0750 (19)	0.0623 (16)	0.0042 (14)	0.0097 (13)	0.0128 (14)

### Geometric parameters (Å, °)

**Table:**

O1—S1	1.444 (2)	C5—C6	1.376 (5)
O2—S1	1.439 (2)	C5—H5	0.9300
N1—H1A	0.8600	C6—S1	1.760 (3)
N1—H1B	0.8600	C7—C8	1.375 (4)
N2—H2B	0.8600	C7—C12	1.386 (4)
N2—H2A	0.8600	C7—S1	1.763 (2)
C1—C6	1.382 (4)	C8—C9	1.401 (3)
C1—C2	1.398 (4)	C8—H8	0.9300
C1—H1	0.9300	C9—N2	1.378 (4)
C2—N1	1.353 (5)	C9—C10	1.390 (4)
C2—C3	1.361 (6)	C10—C11	1.372 (5)
C3—C4	1.365 (6)	C10—H10	0.9300
C3—H3	0.9300	C11—C12	1.386 (4)
C4—C5	1.398 (5)	C11—H11	0.9300
C4—H4	0.9300	C12—H12	0.9300
			
O1—S1—C6	108.32 (13)	C6—C5—C4	118.7 (4)
O1—S1—C7	108.16 (12)	C6—C5—H5	120.6
O2—S1—O1	117.27 (14)	C4—C5—H5	120.6
O2—S1—C6	108.72 (14)	C5—C6—C1	121.7 (3)
O2—S1—C7	108.72 (13)	C5—C6—S1	118.7 (2)
C6—S1—C7	104.97 (12)	C1—C6—S1	119.6 (2)
C2—N1—H1A	120.0	C8—C7—C12	122.6 (2)
C2—N1—H1B	120.0	C8—C7—S1	118.43 (19)
H1A—N1—H1B	120.0	C12—C7—S1	119.0 (2)
C9—N2—H2A	120.0	C7—C8—C9	119.4 (2)
C9—N2—H2B	120.0	C7—C8—H8	120.3
H2B—N2—H2A	120.0	C9—C8—H8	120.3
C6—C1—C2	119.0 (3)	N2—C9—C10	121.3 (2)
C6—C1—H1	120.5	N2—C9—C8	120.5 (3)
C2—C1—H1	120.5	C10—C9—C8	118.2 (3)
N1—C2—C3	120.1 (4)	C11—C10—C9	121.4 (3)
N1—C2—C1	121.2 (4)	C11—C10—H10	119.3
C3—C2—C1	118.7 (3)	C9—C10—H10	119.3
C2—C3—C4	122.9 (4)	C10—C11—C12	120.9 (3)
C2—C3—H3	118.5	C10—C11—H11	119.5
C4—C3—H3	118.5	C12—C11—H11	119.5
C3—C4—C5	119.0 (4)	C7—C12—C11	117.5 (3)
C3—C4—H4	120.5	C7—C12—H12	121.2
C5—C4—H4	120.5	C11—C12—H12	121.2
			
C6—C1—C2—N1	−179.1 (3)	C9—C10—C11—C12	−0.2 (5)
C6—C1—C2—C3	0.0 (4)	C8—C7—C12—C11	0.0 (5)
N1—C2—C3—C4	179.6 (4)	S1—C7—C12—C11	−179.5 (2)
C1—C2—C3—C4	0.4 (6)	C10—C11—C12—C7	−0.1 (5)
C2—C3—C4—C5	−0.8 (7)	C5—C6—S1—O2	−167.8 (2)
C3—C4—C5—C6	0.6 (6)	C1—C6—S1—O2	13.6 (3)
C4—C5—C6—C1	−0.2 (5)	C5—C6—S1—O1	−39.4 (3)
C4—C5—C6—S1	−178.7 (3)	C1—C6—S1—O1	142.1 (2)
C2—C1—C6—C5	−0.1 (4)	C5—C6—S1—C7	76.0 (3)
C2—C1—C6—S1	178.4 (2)	C1—C6—S1—C7	−102.6 (2)
C12—C7—C8—C9	0.4 (4)	C8—C7—S1—O2	144.7 (2)
S1—C7—C8—C9	179.9 (2)	C12—C7—S1—O2	−35.7 (3)
C7—C8—C9—N2	177.2 (3)	C8—C7—S1—O1	16.4 (3)
C7—C8—C9—C10	−0.7 (4)	C12—C7—S1—O1	−164.1 (2)
N2—C9—C10—C11	−177.2 (3)	C8—C7—S1—C6	−99.1 (2)
C8—C9—C10—C11	0.6 (5)	C12—C7—S1—C6	80.4 (3)

### Hydrogen-bond geometry (Å, °)

**Table:**

*D*—H···*A*	*D*—H	H···*A*	*D*···*A*	*D*—H···*A*
N1—H1B···O2^i^	0.86	2.25	3.091 (5)	166
N2—H2A···O1^ii^	0.86	2.29	3.069 (4)	151
N2—H2B···O2^iii^	0.86	2.38	3.187 (4)	156
C1—H1···O2	0.93	2.55	2.924 (4)	104
C8—H8···O1	0.93	2.51	2.895 (3)	105

Symmetry codes: (i) −*x*+1, *y*−1/2, −*z*+1/2; (ii) −*x*, −*y*+1, −*z*; (iii) *x*−1, *y*, *z*.
